# Fish Roe-Induced Anaphylaxis in Italy: A Pediatric Case Report

**DOI:** 10.3390/pediatric14020023

**Published:** 2022-04-08

**Authors:** Nicoletta De Paulis, Margherita Di Costanzo, Maria Elena Capra, Silvia Peveri, Marcello Montagni, Giacomo Biasucci

**Affiliations:** 1 Pediatrics and Neonatology Unit, Department of Maternal and Child Health, Guglielmo da Saliceto Hospital, Azienda USL di Piacenza, 29121 Piacenza, Italy; n.depaulis@usl.pc.it (N.D.P.); m.capra@ausl.pc.it (M.E.C.); g.biasucci@ausl.pc.it (G.B.); 2 Department of Allergology, Guglielmo da Saliceto Hospital, Azienda USL di Piacenza, 29121 Piacenza, Italy; s.peveri@ausl.pc.it (S.P.); m.montagni@ausl.pc.it (M.M.)

**Keywords:** fish roe, salmon roe, lumpfish roe, anaphylaxis, child, food allergy

## Abstract

Fish roe are not yet described as triggers of allergic reactions in Italy, especially during the pediatric age; they are more frequently involved in anaphylaxis in Eastern countries, such as Japan. For this report, we reported a case of anaphylaxis in a 2-year-old boy admitted to our Hospital Pediatric Emergency Room with a suspected allergic reaction. 15 min after the meal, he presented generalized urticaria, angioedema, wheezing, sneezing, and two vomiting episodes. The meal was smoked salmon, butter, mayonnaise, anchovies, and fish roe (salmon and lumpfish roe). Tryptase serum levels presented as elevated in the acute phase and normal after 24 h. Serum food-specific IgE tested negative for salmon and other fish, such as skin prick tests. Serum food-specific IgE showed that the patient was sensitized to cow’s milk and eggs, but he doesn’t have a food allergy. He had regularly consumed milk and eggs before and after the allergic reaction without clinical problems. A prick-by-prick test resulted positive for fish roe (salmon and lumpfish roe). Based on patient’s history, allergy test results in vivo, and tryptase serum levels, the diagnosis of anaphylaxis induced by fish roe was confirmed. In conclusion, to the best of our knowledge, this is the first pediatric case of fish roe-induced anaphylaxis reported in Italy.

## 1. Introduction

Fish roe are not yet described as triggers of allergic reactions in Italy, whereas they are frequently involved in anaphylaxis in Eastern countries, such as Japan, where these foods are more commonly consumed [1,2,3]. Despite this, fish roe seems to be an emerging allergen in Western countries, given the increase in fish roe consumption, especially in sushi dishes. Few cases were recently described in Western countries (three cases in France [4], two cases in the U.S. [5,6], and one case in Portugal [7]). Moreover, most of the case reports on fish roe-induced anaphylaxis refer to adulthood, not to childhood.

Anaphylaxis is defined as a severe, potentially life-threatening systemic allergic reaction, and its diagnosis is based on clinical criteria [8]. In the last three decades, food allergy has been increasing in prevalence, persistence, and severity. The European Anaphylaxis Registry confirmed food as a major elicitor of anaphylaxis in children, specifically hen’s egg, cow’s milk, and nuts [9]. Food items account for approximately half of the patients with anaphylaxis being referred to emergency departments in Western countries [10]. To the best of our knowledge, this is the first pediatric case of fish roe-induced anaphylaxis reported so far in Italy.

## 2. Case Presentation

### 2.1. Medical History

V.R. was a 2-year-old boy when he was referred to our Hospital Pediatric Emergency Room for a suspected allergic reaction. He was born at term via vaginal delivery, with birth weight adequate for gestational age. He had been exclusively breastfed for 6 months; thereafter, complementary feeding, including regular intake of fish, eggs, and cow’s milk, was introduced without clinical problems. His familial history was negative for allergies. At 12 months of age, he was diagnosed with atopic dermatitis and treated with skin moisturizers.

### 2.2. Clinical Presentation

At 2 years of age, due to the occurrence of generalized urticaria, angioedema, wheezing, sneezing, and two vomiting episodes, V.R. was referred to our Hospital Pediatric Emergency Room, after being assisted at home by the territorial Emergency Service Team (EST). On EST first clinical evaluation, he presented with fair general conditions; generalized urticaria, rhinitis, and lips and eyelid angioedema were still present, and his vital signs were as follows: 96% peripheral oxygen saturation rate on ambient air; blood pressure 95/55 mmHg (50° pc 101/63 mmHg); heart rate 150 beats per minute (bpm) (normal range 80–120 bpm), respiratory rate 32 breaths/min (normal range 25 ± 4 breaths/min), and a Glasgow Coma Scale score of 15. The symptoms had occurred 15 min after a meal of smoked salmon, anchovies, mayonnaise, butter, salmon roe, and lumpfish roe. V.R. had previously eaten smoked salmon and mayonnaise without any symptoms. No intake of other foods/juices, alcohol, or medications was reported. During the ambulance trip to the hospital, intramuscular adrenaline (0.01 mg/kg), methylprednisolone (1 mg/kg), and oral cetirizine (0.25 mg/kg) was administered by the EST. The child was admitted to our Pediatric Emergency Department in fair general condition with lips and eyelid angioedema; vital sign parameters were stable compared to the previous evaluation. The results of routine laboratory analysis on admission were within the normal range (Table 1).

### 2.3. Clinical Course

After 12 and 24 h, due to worsening eyelid angioedema with reappearance of urticaria, a further administration of intravenous chlorpheniramine (0.25 mg/kg) was necessary. Tryptase (a protease released from mast cells during an acute allergic reaction) serum level in the acute phase (peak) was 5.39 μg/L, whereas the baseline level, detected 24 h after the allergic reaction, was 2.34 μg/L. In the pediatric population, the peak total serum tryptase should be at least 120% of the baseline tryptase level plus 2 μg/L (≥1.2 × baseline tryptase + 2 μg/L) to diagnose acute mast cell activation in anaphylaxis [11]. Indeed, in our patient, the peak tryptase level met this criterion, exceeding the diagnostic cutoff level (4.81 μg/L = 120% + 2 µg/L of the baseline level). After 36 h of hospitalization, V.R. was discharged in fair clinical condition, though the result of the specific food IgE test was not yet available. An adrenaline auto injection kit (0.15 mg) was prescribed, after instructing the patient of its proper use.

### 2.4. Follow Up and Specific Allergy Tests

One week later, the patient returned to our outpatient office for a routine visit and was in good clinical condition. Food-specific serum IgE (by Immuno-CAP, Thermo Fisher Scientific, Uppsala, Sweden), meantime assessed, tested as follows: egg white (0.67 kUA/L), milk (1.12 kUA/L), beta-lactoglobulin (0.13 kUA/L), caseine (0.18 kUA/L), alfa-lactalbumin (0.48 kUA/L), peanut (<0.35 kUA/L), cod (<0.35 kUA/L), wheat (<0.35 kUA/L), shrimp (<0.35 kUA/L), salmon (<0.35 kUA/L), parvalbumin (<0.35 kUA/L), LTP Pru p3 (<0.35 kUA/L), and Betv2 (<0.35 kUA/L). Level ≥ 0.35 kUA/L was considered positive. Unfortunately, we could not perform the serum food-specific IgE test for fish roe (salmon and lumpfish roe), because it was not yet available in Italy.

**Figure 1:**
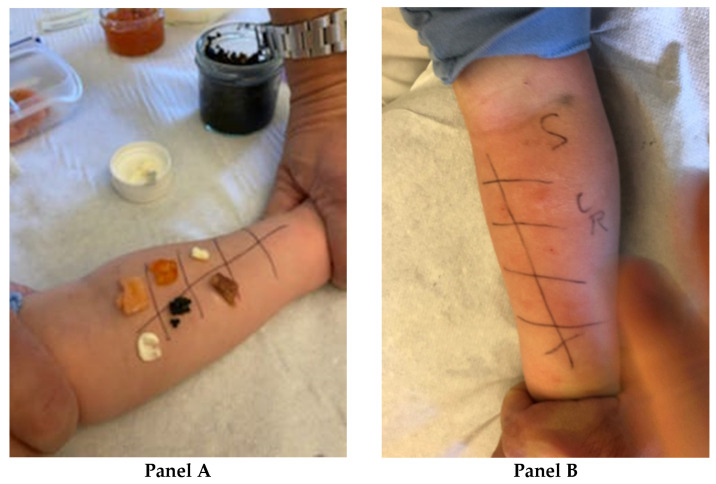
(**Panel A** and **Panel B**). **Prick-by-prick tests performed on our patient.** Prick-by-prick tests were performed for smoked salmon, anchovies, mayonnaise, salmon roe (red caviar), and lumpfish roe (Figure 1, **Panel A**). Prick-by-prick tested positive for salmon roe (red caviar 11 mm × 10 mm) and lumpfish roe (4 mm × 4 mm), but negative for all the other foods tested (Figure 1, **Panel B**). Positive (histamine) and negative (saline solution) controls were included.

Based on a food-specific serum IgE test, only an allergic sensitization to cow’s milk and egg proteins was evident; indeed, these foods had previously been eaten without any symptoms. Prick-by-prick tests for smoked salmon, anchovies, mayonnaise, salmon roe (red caviar), and lumpfish roe were also performed (Figure 1, panel A). All these foods had been eaten a few minutes before the occurrence of the allergic reaction. The prick by-prick test was positive for salmon roe (red caviar 11 mm × 10 mm) and lumpfish roe (4 mm × 4 mm), but negative for all the other foods tested (Figure 1, panel B). Positive (histamine) and negative (saline solution) controls were included.

## 3. Discussion and Conclusions

Based on the patient’s history, allergy test results in vivo, and tryptase serum levels, the diagnosis of anaphylaxis induced by fish roe was confirmed. The diagnosis of anaphylaxis was defined according to the criteria suggested by EAACI Taskforce on the management of anaphylaxis in childhood [8]. In fact, in our patient, we observed an acute onset of the symptoms with involvement of the skin and mucosal tissue and a respiratory compromise. As discussed above, the tryptase serum level in acute phase and at baseline were in line with the clinical diagnosis. The allergy test results finally helped us to define the food which caused the anaphylaxis.

Salmon roe, also known as red caviar or salmon caviar, is routinely eaten in Japan. In Japan, fish roe is the sixth most frequently implicated food in food allergies, even in 2–3-year-old children [1]. In Europe, only few cases have been recently described [4,5,6,7]. Roe from fish or other aquatic species are present within the ovarian membrane and are available from various species of fish, including salmon, cod, herring, pike, smelt, and others; they are often eaten raw in sushi dishes. Roe can vary in terms of size and color, depending on the species. Lipovitellin and β’component are major constituents of yolk proteins in teleost fish roe and have high IgE-binding ability [2,12]. A 35 kDa vitellogenin fragment β’ component is the major allergenic protein of salmon roe; it consists of two identical subunits, named Onc K 5 [13]. No cross-reactivity was found with homologous chicken egg yolk proteins [14]. Cross-reactivity has been proven for roe allergens from different fish species by IgE and skin testing [15,16]. A cross-reactivity with lumpfish roe, identified through the positivity of the skin prick test, was evident in our patient. Given that the skin prick test was positive for both types of fish roe, we cannot doubtlessly define which one of the two allergens is implied, nor if they are both responsible for the reaction; however, considering the recent anaphylaxis, it was not indicated to perform an oral provocation test. There appears to be no cross-reactivity between salmon roe and other salmon muscle [14]. In line with data available in literature so far, our patient had no cross reactivity with fish, specifically salmon and anchovies. He developed a severe reaction to fish roe without concomitant fish allergy. In fact, he was reported to have always eaten fish without any allergic reaction, as supported by negative allergy tests (prick-by-prick test and food specific serum IgE).

In conclusion, children’s food allergy to fish roe is unusual in Western countries, where sushi dish consumption is not so widespread in childhood; however, considering the increased raw fish intake worldwide, the possibility of food allergy to fish roe must be considered in the evaluation of anaphylaxis following the consumption of sushi dishes. Though we are aware that the unavailability of serum-specific IgE concentration for salmon and lumpfish roe may bias our conclusions, we believe that our study may provide further evidence on the need to consider testing even these less common foods during the diagnostic process of anaphylaxis also in the pediatric patients.

## Figures and Tables

**Table 1:** Routine laboratory findings on admission.

	Patient Values	Normal Values
White blood cells (×10^3^/µL)	9.50	6–17
Red blood cells (×10^6^/µL)	5.34	4–5.1
Platelets (×10^3^/µL)	459	150–450
Hemoglobin (g/dL)	14.9	10.3–14.3
Hematocrit (%)	42.5	31–43
Neutrophils (%)	31.3	33
Lymphocytes (%)	61.8	59
Monocytes (%)	4.5	5
Eosinophils (%)	2.1	3
C-reactive protein (mg/dL)	0.03	0–0.5
Glycemia (mg/dL)	112	74–100
Urea (mg/dL)	36	10–50
Creatinine (mg/dL)	0.31	0.6–1.2
Sodium (mEq/L)	141	135–146
Potassium (mEq/L)	4.3	3.6–5
Chlorine (mEq/L)	106	97–110
Calcium (mg/dL)	10.40	8.10–10.40
Aspartate aminotransferase (U/L)	41	10–50
Alanine aminotransferase (U/L)	34	15–40
LDH (U/L)	391	150–500

## Data Availability

Not applicable.
